# Residency Experience With Physical Examination– and Ultrasound-Indicated Cerclage: A Single Center Retrospective Study

**DOI:** 10.31486/toj.22.0092

**Published:** 2023

**Authors:** Burk Schaible, Darcy Langhals, Lesli Taylor, Jennifer Gold, Dara Seybold, Byron C. Calhoun

**Affiliations:** ^1^ Department of Obstetrics and Gynecology, West Virginia University–Charleston Division, Charleston Area Medical Center, Charleston, WV; ^2^ Center for Health Services and Outcomes Research, CAMC Institute for Academic Medicine, Charleston, WV; ^3^ West Virginia University School of Medicine, Charleston Campus, Charleston, WV

**Keywords:** *Cervical–cerclage*, *intensive care–neonatal*, *perinatal mortality*, *premature birth*

## Abstract

**Background:** Cervical insufficiency, the dilation of the cervix in the absence of contractions or labor, can cause second-trimester pregnancy loss or preterm birth. Cervical cerclage is a common treatment for cervical insufficiency and has 3 indications for placement: history, physical examination, and ultrasound. The purpose of this study was to compare pregnancy and birth outcomes for physical examination– and ultrasound-indicated cerclage.

**Methods:** We conducted a retrospective observational descriptive review of second-trimester obstetric patients with transcervical cerclage performed by residents at a single tertiary care medical center from January 1, 2006, to January 1, 2020. We present data on all patients and compare outcomes between the 2 study groups: patients who received physical examination–indicated cerclage vs those who received ultrasound-indicated cerclage.

**Results:** Cervical cerclage was placed on 43 patients at a mean gestational age of 20.4 ± 2.4 weeks (range, 14 to 25 weeks) and with a mean cervical length of 1.53 ± 0.5 cm (range, 0.4 to 2.5 cm). With a latency period of 11.8 ± 5.7 weeks, mean gestational age at delivery was 32.1 ± 6.2 weeks. Fetal/neonatal survival rates were comparable: 80% (16/20) for the physical examination group compared to 82.6% (19/23) for the ultrasound group. No differences were found between groups for gestational age at delivery—31.5 ± 6.8 in the physical examination group vs 32.6 ± 5.8 in the ultrasound group (*P*=0.581)—or for preterm birth <37 weeks—65.0% (13/20) in the physical examination group vs 65.2% (15/23) in the ultrasound group (*P*=1.000). Rates of maternal morbidity and neonatal intensive care unit morbidity were similar between the groups. No cases of immediate operative complications or maternal deaths occurred.

**Conclusion:** Pregnancy outcomes for physical examination– and ultrasound-indicated cerclage placed by residents at a tertiary academic medical center were similar. Fetal/neonatal survival and preterm birth rates were favorable for physical examination–indicated cerclage when compared to other published studies.

## INTRODUCTION

Cervical insufficiency, a condition in which the cervix dilates and effaces in the second trimester without contractions or labor, can lead to pregnancy loss or preterm birth. Preterm birth is a major cause of neonatal morbidity and mortality in the United States.^1^ Current modalities for cervical insufficiency include progesterone supplementation, cervical length screening, and cervical cerclage placement. The 3 indications for cerclage are history, physical examination, and ultrasound.^2-4^ Although national guidelines for history-indicated cerclage vary, this prophylactic treatment is indicated for females who have a history of midtrimester pregnancy loss or preterm delivery.^2^ Physical examination–indicated cerclage, also called rescue or emergency cerclage, is indicated when cervical changes occur, such as dilation of 1 cm or more, or prolapsed fetal membranes are visible during a physical examination with a speculum followed by digital palpation.^1^ Ultrasound-indicated cerclage is placed in patients with a history of spontaneous loss or preterm delivery with a cervical length <2.5 cm per transvaginal scan before 24 weeks’ gestation.^2,3^

Efficacy varies by cerclage indication. History-indicated cerclage has been assessed in 3 randomized controlled trials. Two of the trials found no significant differences in outcomes between the no-cerclage and cerclage groups,^5,6^ while the other trial reported a statistically significant reduction in preterm births <33 weeks in patients receiving cerclage (13%) vs controls (17%, *P*=0.03).^7^ For ultrasound-indicated cerclage, a meta-analysis of trials involving patients with singleton pregnancies concluded that cerclage placement was associated with a significant decrease in the risk of preterm birth before 35 weeks of gestation: 28% in the cerclage group compared with 41% in the no-cerclage group.^8^ While few randomized studies have adequate power to strongly support or refute the use of physical examination–indicated cerclage, 2 meta-analyses suggest superior outcomes with cerclage placement compared to expectant management in patients with physical examination–indicated cerclage.^9,10^ In the absence of strong data, the American College of Obstetricians and Gynecologists (ACOG) considers physical examination–indicated cerclage a reasonable treatment after careful consideration of the risks and benefits.^3^

While the risk of complications from cerclage is relatively low, cerclage in the presence of membrane rupture or dilation is associated with increased risk.^3^ Thus, physical examination–indicated cerclage could be considered to have a higher risk for complications than ultrasound- or history-indicated cerclage. In addition, physical examination–indicated cerclage has been associated with prelabor rupture of membranes.^11^ Cerclage in cases of twin pregnancy may increase the risk of preterm birth.^4^ ACOG recommends offering cervical cerclage after placental abruption, labor, and intra-amniotic infection are ruled out.^3^ Cerclage may potentially prolong pregnancy and thereby reduce neonatal morbidity and mortality associated with preterm birth. The question is whether the pregnancy and birth outcomes of physical examination–indicated cerclage are similar to those of ultrasound-indicated cerclage. Thus, the purpose of this study was to compare maternal and fetal/neonatal outcomes of physical examination–indicated cerclage and ultrasound-indicated cerclage performed by senior resident physicians under attending physician guidance at a single tertiary care center.

## METHODS

This retrospective observational descriptive review of patients was conducted at Charleston Area Medical Center (CAMC), a single university-affiliated tertiary care center located in Charleston, West Virginia, from January 1, 2006, to January 1, 2020. The CAMC/West Virginia University Institutional Review Board approved this study (study number 20-673). Study staff complied with the World Medical Association Declaration of Helsinki regarding ethical conduct of research involving human subjects. Written patient consent was not required given the retrospective nature of the study and deidentified patient characteristics. No external funding was obtained for this study.

Second-trimester obstetric patients with cerclage placement were identified with International Classification of Diseases code 034.3. Medical records were reviewed, and all patients were confirmed to have cerclage placed for either a physical examination or ultrasound indication, with a resident as the primary surgeon under attending physician supervision.

History-indicated cerclage is typically placed at 12 to 14 weeks’ gestation and is technically easier than physical examination–indicated and ultrasound-indicated cerclage because of the absence of cervical dilation, longer cervical length, and the absence of prolapsing membranes. Physical examination–indicated cerclage and ultrasound-indicated cerclage are more technically challenging because of advanced gestational age (typically placed at 16 to 24 weeks’ gestation), short or dilated cervix, and prolapsing membranes. To examine the impact of resident involvement with more difficult cerclage placement, cases of history-indicated cerclage were excluded from this study.^12,13^ Cerclage was not offered to patients with suspected uterine infection diagnosed by standard clinical criteria, preterm premature rupture of membranes, or lethal fetal anomalies. Amniocentesis was not performed on any patient.

### Procedure

Preoperative evaluation was standardized for all patients undergoing cerclage and included fetal anatomic survey and screening for signs or symptoms of maternal infection: maternal fever, abdominal tenderness, or abnormal discharge. Laboratory assessment included testing for bacterial vaginosis, chlamydia, and gonorrhea; urinalysis with urine culture; aneuploidy screening (when available); and complete blood count with differential and platelet count. After a discussion with the patient about the known risks and benefits of cervical cerclage, informed written and verbal consents were obtained prior to the cerclage procedure. Preoperative cefazolin was used as antimicrobial prophylaxis unless contraindicated based on patient allergy. Clindamycin was used alternatively in these cases. Unless specific contraindications were encountered, standardized operative techniques were used: spinal anesthesia, lithotomy position with Trendelenburg as needed, Foley catheter placement with bladder distention of 300 cc of sterile fluid (to elevate fetal membranes), vaginal preparation with iodine, McDonald cerclage in usual purse-string fashion with 5-mm Mersilene braided suture tied at 12 o’clock, and postprocedure irrigation with a liter of sterile fluid. Indomethacin was given postoperatively for 24 hours (100 mg twice daily by mouth loading dose and 25 mg by mouth for 3 doses every 6 hours) with 1 gm sucralfate (Carafate) for gastric protection. A tocodynamometer was placed, and patients were observed overnight or longer if needed. Suture was removed at 36 to 37 weeks’ gestation in clinic or earlier if clinically indicated.

### Data and Statistical Analysis

Patient demographic information, obstetric history, characteristics of the current pregnancy, and fetal/neonatal outcomes were obtained through the institutional data warehouse and abstracted from electronic medical records. Clinical characteristics of cerclage were also assessed. The following outcomes are reported: number of weeks of latency (time period between cerclage placement and delivery), mode of delivery (vaginal or cesarean), maternal morbidity, gestational age at delivery, preterm birth, incidence of intrauterine fetal demise and neonatal mortality, neonatal intensive care unit (NICU) admission, gestational age at NICU admission, NICU length of stay, and neonatal morbidity.

Neonatal morbidity was defined as having 1 or more of the following: intraventricular hemorrhage, respiratory distress syndrome, bronchopulmonary dysplasia, sepsis or other infection, retinopathy of prematurity, necrotizing enterocolitis, or pulmonary hypertension. Maternal morbidity was defined as having 1 or more of the following: infection, operative complication, need for blood products, or postpartum readmission. Operative complication was defined as having any of the following: intraoperative bleeding >100 mL, intraoperative rupture of membrane, chorioamnionitis, anesthesia complications, or displacement of the suture.

Data were stratified by cerclage indication: physical examination vs ultrasound. Statistical analysis was conducted with SPSS Statistics for Windows, v. 19 (IBM Corporation). We used Fisher exact test or Pearson chi-squared test for categorical variables and report them as frequencies and percentages. For continuous variables, we compared mean values with independent sample *t* test and report as means ± standard deviations. Statistical significance was defined as *P*<0.05. Kaplan-Meier survival analysis was performed to compare weeks from cerclage until delivery (latency) between the physical examination and ultrasound groups.

## RESULTS

**Figure 1.**
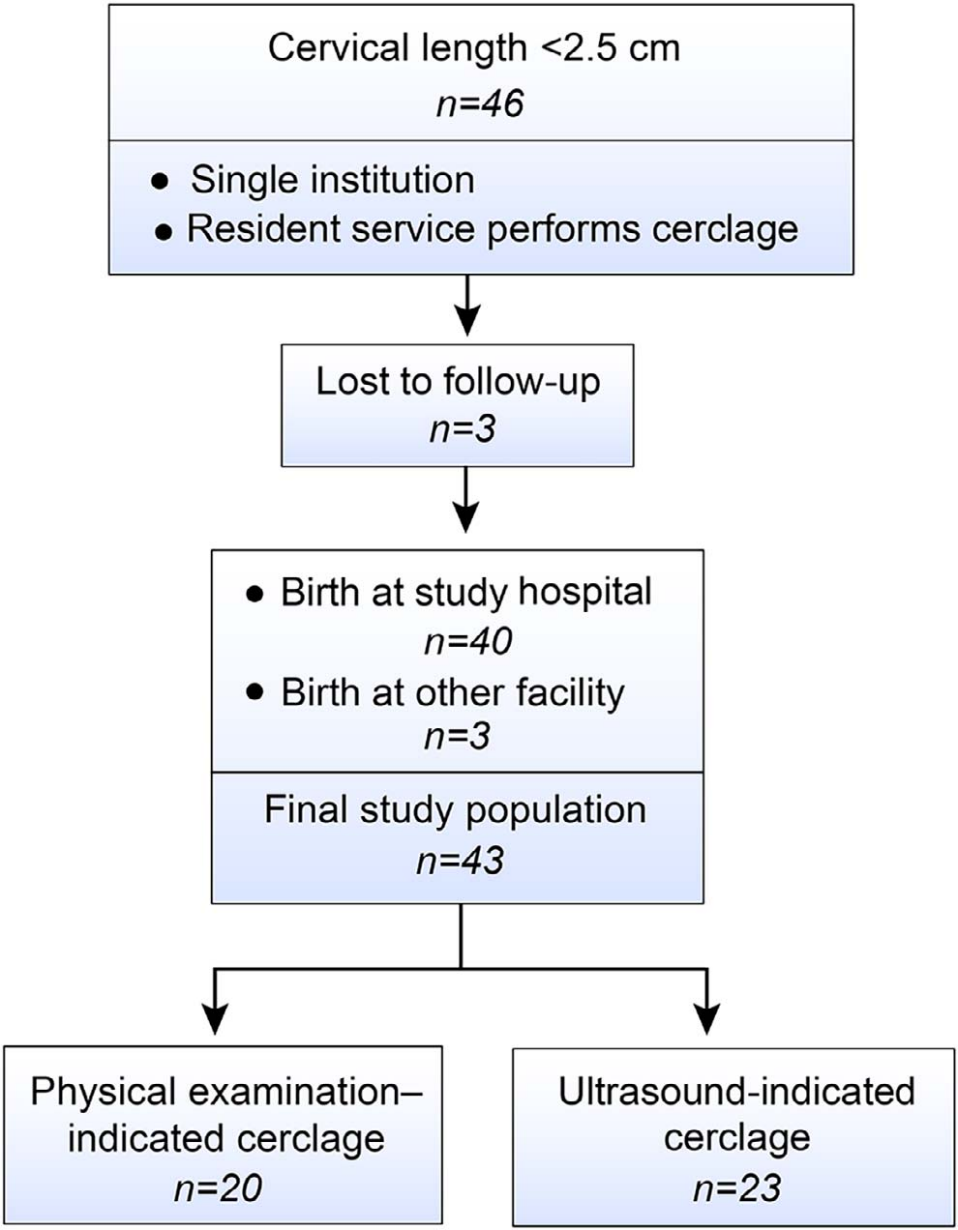
Study population.

Forty-six patients were identified as having cerclage placement by the resident service during the study period. While 6 patients delivered outside our institution, delivery data were successfully collected on 3 patients through request of records. Thus, the final sample size was 43 patients (Figure 1), with 20 in the physical examination–indicated cerclage group and 23 in the ultrasound-indicated cerclage group. Maternal characteristics of the total study population and by cerclage indication are presented in Table 1. The only statistically significant differences between the 2 groups were nulliparity and gravidity. Although the ultrasound group had higher rates of preexisting diabetes, hypertension, depression, and tobacco and substance use history, the differences between the groups were not statistically significant.

**Table 1.** Maternal Characteristics by Cerclage Indication

Characteristic	All Patients, n=43	Physical Examination–Indicated Cerclage, n=20	Ultrasound-Indicated Cerclage, n=23	*P* Value
Age, years, mean ± SD	27.4 ± 6.4	27.6 ± 6.0	27.0 ± 6.0	0.742
Race/ethnicity				0.346
White/Caucasian	35 (81.4)	17 (85.0)	18 (78.3)	
Black/African American	7 (16.3)	2 (10.0)	5 (21.7)	
Hispanic	1 (2.3)	1 (5.0)	0 (0.0)	
Gravidity, mean ± SD	3.4 ± 2.0	2.5 ± 2.0	4.3 ± 1.6	0.002
Nulliparous	10 (23.3)	10 (50.0)	0 (0.0)	<0.001
Comorbidities				
Preexisting diabetes	1 (2.3)	0 (0.0)	1 (4.3)	0.575
Hypertension	4 (9.3)	1 (5.0)	3 (13.0)	0.610
Depression	9 (20.9)	2 (10.0)	7 (30.4)	0.142
History of tobacco use	25 (58.1)	11 (55.0)	14 (60.9)	0.763
History of substance use	16 (37.2)	6 (30.0)	10 (43.5)	0.528

Note: Data are presented as n (%) unless otherwise indicated.

Obstetric history and current pregnancy information are presented in Table 2. Twin pregnancies occurred in 18.6% of the study population. Rates of prior preterm birth and/or pregnancy loss and of progesterone supplement use were statistically higher in the ultrasound group. Only patients in the physical examination group had sexually transmitted infections (15%).

**Table 2.** Obstetric History and Characteristics of Current Pregnancy

Variable	All Patients, n=43	Physical Examination–Indicated Cerclage, n=20	Ultrasound-Indicated Cerclage, n=23	*P* Value
History				
Prior preterm birth and/or pregnancy loss	31 (72.1)	8 (40.0)	23 (100.0)	<0.001
Dilation and curettage	21 (48.8)	8 (40.0)	13 (56.5)	0.364
Progesterone supplement	7 (16.3)	0 (0.0)	7 (30.4)	<0.001
Cervical surgery	7 (16.3)	4 (20.0)	3 (13.0)	0.678
Current pregnancy				
Twin gestation	8 (18.6)	4 (20.0)	4 (17.4)	1.000
Vaginal infection	21 (48.8)	8 (40.0)	13 (56.5)	0.364
Group B streptococcus	6 (14.0)	3 (15.0)	3 (13.0)	1.000
Urinary tract infection	8 (18.6)	4 (20.0)	4 (17.4)	1.000
Sexually transmitted infection	3 (7.0)	3 (15.0)	0 (0.0)	0.092
Tobacco use	14 (32.6)	5 (25.0)	9 (39.1)	0.353
Substance use	16 (37.2)	6 (30.0)	10 (43.5)	0.342

Note: Data are presented as n (%).

**Figure 2.**
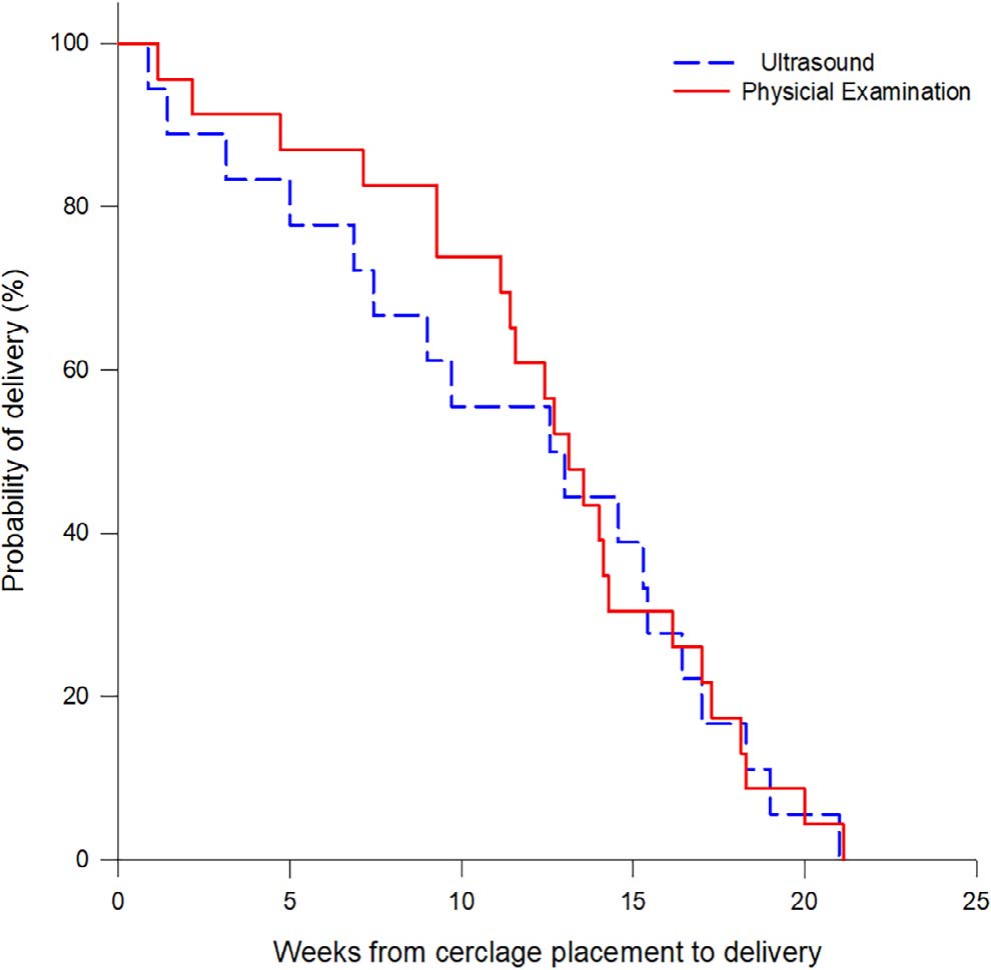
Kaplan-Meier survival curve shows little difference in latency between the physical examination and ultrasound groups after 13 weeks.

Characteristics of cerclage are reported in Table 3. Cerclage was placed in the 2 groups at similar gestational ages (20.4 ± 2.4 weeks overall). Cervical lengths ranged from 0.4 cm to 2.5 cm. Mean cervical length at cerclage and latency, or weeks from cervical placement to delivery, were not statistically different. Figure 2 shows that in the weeks immediately after cerclage, pregnancies lasted longer in the physical examination group than the ultrasound group, but little difference in latency was seen between the 2 groups after 13 weeks.

**Table 3.** Cerclage Characteristics

Characteristic	All Patients, n=43	Physical Examination–Indicated Cerclage, n=20	Ultrasound-Indicated Cerclage, n=23	*P* Value
Gestational age at cerclage, weeks, mean ± SD	20.4 ± 2.4	20.9 ± 2.0	19.9 ± 2.6	0.198
Cervical length at cerclage, cm, mean ± SD	1.53 ± 0.5	1.6 ± 0.4	1.4 ± 0.5	0.102
Prophylactic antibiotic	36 (83.7)	17 (85.0)	19 (82.6)	1.000
Indomethacin postoperatively	32 (74.4)	15 (75.0)	17 (73.9)	1.000

Notes: Data are presented as n (%) unless otherwise indicated.

For pregnancy outcomes, no significant differences were found between the physical examination and ultrasound groups (Table 4). Rates for preterm birth were comparable, although the physical examination group had higher preterm birth rates than the ultrasound group at the <35, <32, and <28 weeks’ gestation time points.

**Table 4.** Maternal and Fetal Birth Outcomes by Cerclage Indication

Outcome	All Patients, n=43	Physical Examination–Indicated Cerclage, n=20	Ultrasound-Indicated Cerclage, n=23	*P* Value
**Maternal/Fetal**				
Latency, weeks, mean ± SD^a^	11.8 ± 5.7	11.0 ± 6.1	12.6 ± 5.4	0.314
Cesarean section	12 (27.9)	7 (35.0)	5 (21.7)	0.265
Maternal morbidity	10 (23.3)	4 (20.0)	6 (26.1)	0.495
Gestational age at delivery, weeks, mean ± SD	32.1 ± 6.2	31.5 ± 6.8	32.6 ± 5.8	0.581
Preterm birth				
<37 weeks	28 (65.1)	13 (65.0)	15 (65.2)	1.000
<36 weeks	26 (60.5)	12 (60.0)	14 (60.9)	1.000
<35 weeks	24 (55.8)	12 (60.0)	12 (52.2)	0.760
<32 weeks	18 (41.9)	9 (45.0)	9 (39.1)	0.544
<28 weeks	14 (32.6)	8 (40.0)	6 (26.1)	0.515
Intrauterine fetal demise	4 (9.3)	2 (10.0)	2 (8.7)	1.000
**Neonatal**	**n=39**	**n=18**	**n=21**	
Neonatal mortality	4 (10.3)	2 (11.1)	2 (9.5)	1.000
NICU outcomes	**n=36**	**n=16**	**n=20** **^b^**	
NICU admission	19 (52.8)	9 (56.3)	10 (50.0)	0.749
Gestational age at NICU admission, weeks, mean ± SD	33.8 ± 5.0	33.9 ± 5.1	33.7 ± 5.0	0.901
NICU length of stay, days, mean ± SD	24.5 ± 40.0	25.8 ± 39.5	23.6 ± 41.3	0.872
NICU morbidity	13 (36.1)	6 (37.5)	7 (35.0)	1.000
Respiratory distress syndrome	12 (33.3)	5 (31.3)	7 (35.0)	0.726
Bronchopulmonary dysplasia	8 (22.2)	4 (25.0)	4 (20.0)	1.000
Infection including sepsis	7 (19.4)	4 (25.0)	3 (15.0)	0.650
Retinopathy of prematurity	5 (13.9)	2 (12.5)	3 (15.0)	1.000
Necrotizing enterocolitis	3 (8.3)	2 (12.5)	1 (5.0)	0.600
Pulmonary hypertension	3 (8.3)	1 (6.3)	2 (10.0)	1.000

^a^Latency is the number of weeks from cerclage placement to delivery.

^b^This group includes 1 neonate who died in the NICU.

Note: Data are presented as n (%) unless otherwise indicated.

NICU, neonatal intensive care unit.

No maternal deaths occurred. While no immediate operative complications occurred, the overall maternal morbidity rate of 23.3% (10/43) included 9 (20.9%) patients with vaginal infections. Some patients had more than 1 morbidity; 1 patient (2.3%) required a blood transfusion later during pregnancy, and 2 patients (4.7%) were readmitted to the hospital during the postpartum period for conditions unrelated to cerclage placement. The neonatal survival rate was 81.4% (35/43)—with 4 intrauterine fetal demises and 4 neonatal deaths (including 1 in the NICU)—and was similar for the physical examination and ultrasound groups. Of the 36 live neonates, more than half (19/36, 52.8%) were admitted to the NICU. The average gestational age for NICU admission was 33.8 ± 5.0 weeks, and 36.1% (13/36) neonates experienced morbidity. No differences in either maternal/fetal or neonatal outcomes were seen between the physical examination and ultrasound groups.

## DISCUSSION

In our study, 67.4% of patients delivered after 28 weeks, and the neonatal survival rate was 81.4% (35/43). While we did not have a control group, these outcomes compare favorably with similar studies. In a retrospective study of 74 patients, Gundabattula et al reported that 42% of patients delivered after 28 weeks and the neonatal survival rate was 50.7%.^14^ A widely cited systematic review and meta-analysis that included 10 studies (randomized trials and cohort studies) reported that 485 of 757 (64%) patients underwent physical examination–indicated cerclage compared to 272 (36%) who were expectantly managed.^9^ Cerclage placement resulted in a 71% neonatal survival rate compared with 43% for expectant management. Nelson et al reported that 23.5% of patients with physical examination–indicated cerclage gave birth beyond 36 weeks’ gestation.^15^ Our study results in the physical examination group were better, with 40% (8/20) delivering after 36 weeks as reported in Table 4. In their study comparing the 3 indications for cerclage, Chen et al showed that history- and ultrasound-indicated cerclage had similar outcomes, but the physical examination–indicated cerclage group had statistically significant differences in gestational age at delivery and fetal survival rate, with a 40.0% fetal survival rate.^16^ Our study showed results similar to Gluck et al as their study showed no difference between emergency (physical examination–indicated) and elective (ultrasound-indicated) cerclage for birth beyond 37 weeks’ gestation.^17^ No maternal deaths occurred in our study population.

Our study has several strengths. To our knowledge, this study is one of few evaluations of maternal and fetal/neonatal outcomes after rescue cerclage performed by residents as the primary operating surgeon during a multiyear training period (2006 to 2020). Another strength is the uniformity of this study. All patients were treated under the same surgical protocol, including retrograde filling of the bladder and use of the McDonald technique with Mersilene suture, and the majority of patients received antibiotics and tocolytics. The tocolytic agent, indomethacin, was given postoperatively up to 24 hours after cerclage. Almost all patients delivered at our center (40 of 46 patients), allowing us to access all delivery and neonatal data. Each case involved a senior resident (third- or fourth-year obstetrics and gynecology resident) under the supervision of a single maternal fetal medicine physician. When compared to similar studies, cerclage performed in our study did not notably increase maternal or fetal/neonatal patient morbidity or mortality. By excluding cases of history-indicated cerclage, heterogeneity was avoided that could have artificially inflated the mean latency period from the time of cerclage placement to delivery.

The limitations of this study include a single site with a relatively homogenous population, making our findings less generalizable; however, the comorbidities of the population may mitigate this issue because of the high rates of history of tobacco use (58.1%) and polysubstance use (37.2%). Nevertheless, our findings may not be generalizable to all tertiary academic medical centers. The other limitations of the study are the retrospective nature and small number of patients involved. Last, we did not include a control group because of the inability to control for all maternal and neonatal variables. Given the data in support of physical examination– and ultrasound-indicated cerclage, randomizing treatment and withholding cerclage for patients with a clear indication would be ethically challenging. A potential control group would be patients refusing cerclage placement in favor of expectant management. However, given the relatively rare nature of this clinical scenario, obtaining enough patients to make meaningful clinical conclusions would be difficult.

Future studies should consider randomizing aspects of the procedure such as suture material, antibiotics, tocolytics, and/or progesterone to assess the individual effect these variables have on cerclage placement. Until these studies are complete, our study shows that our technique is safe and effective for patients with either physical examination– or ultrasound-indicated cerclage.

## CONCLUSION

Pregnancy outcomes for physical examination– and ultrasound-indicated cerclage placed by residents at a tertiary academic medical center were similar. Our fetal/neonatal survival and preterm birth rates were favorable for physical examination–indicated cerclage when compared to other published studies.

## Notes

*This article meets the Accreditation Council for Graduate Medical Education and the American Board of Medical Specialties Maintenance of Certification competencies for Patient Care, Medical Knowledge, and Practice-Based Learning and Improvement.*
